# What About My Weight? Insufficient Weight Loss or Weight Regain After Bariatric Metabolic Surgery

**DOI:** 10.5812/ijem-136329

**Published:** 2023-11-08

**Authors:** Hamidreza Zefreh, Reza Amani-Beni, Erfan Sheikhbahaei, Farnaz Farsi, Shahrzad Ahmadkaraji, Maryam Barzin, Bahar Darouei, Alireza Khalaj, Shahab Shahabi

**Affiliations:** 1 Minimally Invasive Surgery and Obesity Research Center, School of Medicine, Alzahra University Hospital, Isfahan University of Medical Sciences, Esfahan, Iran; 2 School of Medicine, Isfahan University of Medical Sciences, Isfahan, Iran; 3 Cardiac Rehabilitation Research Center, Cardiovascular Research Institute, Isfahan University of Medical Sciences, Isfahan, Iran; 4 Minimally Invasive Surgery Research Center, Hazrat-E Rasool General Hospital, Iran University of Medical Sciences, Tehran, Iran; 5 Obesity Research Center, Research Institute for Endocrine Sciences, Shahid Beheshti University of Medical Sciences, Tehran, Iran; 6 Department of Surgery, School of Medicine, Tehran Obesity Treatment Center, Shahed University, Tehran, Iran

**Keywords:** Insufficient Weight Loss, Weight Regain, Bariatric Surgery, Disease Management, Prevention and Control

## Abstract

**Context:**

This review study aimed to investigate the definition, etiology, risk factors (RFs), management strategy, and prevention of insufficient weight loss (IWL) and weight regain (WR) following bariatric metabolic surgery (BMS).

**Evidence Acquisition:**

Electronic databases were searched to retrieve relevant articles. The inclusion criteria were English articles with adult participants assessing the definition, prevalence, etiology, RFs, management strategy, and prevention of IWL/WR.

**Results:**

Definition: The preferred definition for post-BMS IWL/WR are the terms "Lack of maintenance of total weight loss (TWL)>20%" and "weight change in percentage compared to nadir weight or weight loss". Prevalence: The exact prevalence of IWL/WR is still being determined due to the type of BMS and various definitions. Etiology: Several mechanisms, including hormonal/metabolic, dietary non-adherence, physical inactivity, mental health, and anatomic surgical failure, are possible etiologies of post-BMS IWL/WR. Risk factors: Preoperative body mass index (BMI), male gender, psychiatric conditions, comorbidities, age, poor diet, eating disorders, poor follow-ups, insufficient physical activity, micronutrients, and genetic-epigenetic factors are the most important RFs. Management Strategy: The basis of treatment is lifestyle interventions, including dietary, physical activity, psychological, and behavioral therapy. Pharmacotherapy can be added. In the last treatment line, different techniques of endoscopic surgery and revisional surgery can be used. Prevention: Behavioral and psychotherapeutic interventions, dietary therapy, and physical activity therapy are the essential components of prevention.

**Conclusions:**

Many definitions exist for WR, less so for IWL. Etiologies and RFs are complex and multifactorial; therefore, the management and prevention strategy is multidisciplinary. Some knowledge gaps, especially for IWL, exist, and these gaps must be filled to strengthen the evidence used to guide patient counseling, selection, and improved outcomes.

## 1. Context

Bariatric metabolic surgery (BMS) has been established as the most efficacious and durable intervention for lowering body weight, maintaining weight loss in severe obesity, and improving or remission in comorbidities (1). Unfortunately, in a variable portion of patients, insufficient weight loss (IWL) and weight regain (WR) can occur following BMS, which are the common reasons to qualify for revisional BMS (2).

As we know, obesity has become like an epidemic, and as a result, more individuals are undergoing bariatric procedures as a form of treatment. In order to avoid patient discontent, poor quality of life following surgery, and the recurrence of associated disorders, it is crucial to understand IWL and WR thoroughly. Therefore, to address knowledge gaps and understand these two complications, this review aimed to have an overview of the definition, prevalence, etiology, risk factors (RFs), management strategy, and prevention of IWL and WR following BMS.

## 2. Evidence Acquisition

Two authors (R. A. and H. Z.) conducted a search in PubMed, Scopus, EMBASE, Web of Science, Google Scholar, and Cochrane electronic databases in order to retrieve English articles published up to February 2023 using the combination of the following medical search terms (“insufficient weight loss”, “weight regain”, “definition”, “etiology”, “mechanism”, “predictor”, “risk factor”, “management”, “diet*”, “behavior*”, “lifestyle”, “physical activity”, “exercise”, “drug”, “surgery”, and “prevention”). The authors also screened the bibliographic references of relevant articles and existing reviews by hand-searching. The titles and abstracts of studies were independently screened for duplication using Endnote software (version 20). Different studies were reviewed, including observational studies, clinical trials, and reviews relevant to the subjects.

The inclusion criteria were articles assessing the definition, prevalence, etiology, RFs, management strategy, and prevention of IWL and WR. Information related to these topics was also extracted from the articles. Only the studies with participants over 18 years (adults) were included. Studies had to be available in English.

Non-human studies, conference papers and abstracts, errata, commentaries, editorials, and studies that did not provide accurate and clear data or methods were excluded. Any disagreement was resolved by consensus and the third author’s final decision (Sh. Sh.). The final selection of papers was made based on their relevance and confirmed by all authors.

## 3. Results

### 3.1. Definition

Although several studies have defined IWL and WR, a consensus definition has yet to be accepted as the standard (Table 1). This issue creates a barrier to determining the effective treatment and difficulty in comparing different studies (3). Insufficient weight loss has fewer assessed definitions than WR. Although excess weight loss (EWL) is highly popular and can be generalized to a wide range of articles, total weight loss (TWL) has recently been used more in BMS reports. Additionally, the definition based on TWL as “lack of maintenance of TWL > 20%” is preferable since it has a stronger significant correlation with clinical outcomes (4).

**Table 1.** Published Definitions of Insufficient Weight Loss (IWL) and Weight Regain (WR) After Bariatric Surgery

IWL/WR	Unit	Definitions
**IWL**	TWL	< 20% TWL, over time (4)
	EWL	EWL < 50%, 18 months postoperative (5)
		EWL < 50%, from preoperative weight (6)
	Other	Primary nonresponse: Inability to achieve adequate weight loss after surgery (7)
**WR**	TWL	Lack of maintenance of TWL > 20% (8)
	EWL	EWL < 50% after reaching EWL > 50% (5)
		> 15% of maximal EWL (9)
		> 25% EWL from nadir weight (10)
		REWL (best postoperative EWL - current measured EWL) > 25% (11)
	BMI	≥ 5 BMI points from nadir weight (12)
		BMI ≥ 35 kg/m^2^ after successful weight loss (10)
	EWL+BMI	BMI ≥ 30 + EWL < 50% (13)
		BMI ≥ 35 + EWL < 50% (14)
	kg	≥ 5 kg from nadir weight, two-year status post-sleeve gastrectomy (15)
		> 10 kg weight gain from lowest postoperative weight (15)
		≥ 10 kg from nadir weight (16)
	Percentage	Percentage of weight regained (mild 0.5%, moderate 0.5 - 1%, and severe 1%) over nadir weight, 30 days from nadir (17)
		≥ 10% total weight from nadir (18)
		> 15% total weight from nadir (19)
		Relative to the amount of weight loss (20)
		> 10% of the lowest postoperative weight, two-year status post-Roux-en-Y gastric bypass (21)
		≥ 10% of the lowest postoperative weight (18)
		> 15% of the lowest postoperative weight (22)
		≥ 20% of the lowest postoperative weight (18)
		≥ 25% of the lowest postoperative weight (18)
		≥ 10% of preoperative weight (18)
	Others	Any WR, especially after remission of type 2 diabetes (10); Change in BMI, TWL, excess BMI lost, EWL from the nadir weight, 5 years postoperatively (10)
		Progressive weight regain that occurs after the achievement of an initially successful weight loss defined as EWL > 50% (23)
		Secondary nonresponse: Excessive WR after initial adequate weight loss after surgery (7)
		Progressive WR after an initial successful weight loss (EWL > 50%) (23)
		WR percentage = (5-year recorded weight - minimum recorded weight × 100) / (preoperative weight - minimum recorded weight) (10)

Abbreviations: IWL, insufficient weight loss; WR, weight regain; TWL, total weight loss; EWL, excess weight loss; REWL, relative excess weight loss; BMI, body mass index; kg, kilogram.

On the other hand, WR has a broad range of definitions. As mentioned for IWL, the definition based on TWL is preferred (8). Body mass index (BMI) is less frequently used for defining WR or IWL. Additionally, an increase in BMI or BMI ≥30 kg/m^2^ has not been correlated with comorbidities recurrence (3). “Any WR” definitions are also not preferable, making the definition too broad to even include patients with maintaining a proper resolution of the comorbidities. It is also considered normal to have slight weight fluctuation, even in patients who have reached their goal (16).

Weight-based definitions of WR, including weight change in kilogram (kg) and percentage, are very constrained (16). There is no standard cutoff for weight rise, such as a five kg increase from the lowest postoperative weight. Any value would not be standard due to no clinically significant established weight (15, 16). Additionally, using a weight change in percentage units is more relevant. This percentage is usually compared to the nadir weight, the lowest postoperative or preoperative weight, and the amount of weight loss (17, 18, 20). Although no standard definition of nadir weight and WR related to weight loss is agreed on, recently published articles have used these definitions more frequently (8, 12). Moreover, two definitions of WR, ≥10 kg rise from nadir weight and >15% increase from nadir weight, are among the most common and widely used definitions. Weight regain change in percentage measured relative to the weight preoperatively is also recommended (18). Further studies are required to investigate the association between these definitions and BMS results and the improvement in comorbidities.

### 3.2. Prevalence

The exact prevalence of IWL and WR is still being determined due to low follow-up rates, the type of BMS performed, and various definitions. Overall, studies using higher WR cutoffs showed a difference in WR of between 23.7% (21) and 38.33% (24); nevertheless, studies using lower WR cutoffs showed a difference in WR of between 39.3% (25) and 59.6% (26).

Differences in surgical procedures impacting WR were 38% for post-laparoscopic adjustable gastric banding (LAGB) (27), 27.8% for post-laparoscopic sleeve gastrectomy (LSG) (28), and 3.9% for post-Roux‐en‐Y gastric bypass (RYGB) (29). In a study by Conceicao et al., there was a significant association between the type of BMS and the prevalence of WR; accordingly, the prevalence of WR in LAGB was 17.7%; in comparison, this prevalence was 5.5% for laparoscopic RYGB (30). As for IWL, the prevalence of WR after LSG (31, 32) and after RYGB, one-anastomosis gastric bypass (OAGB), and LSG combined (33) were 32 - 40% and 20%, respectively.

### 3.3. Etiology

**Figure 1.**
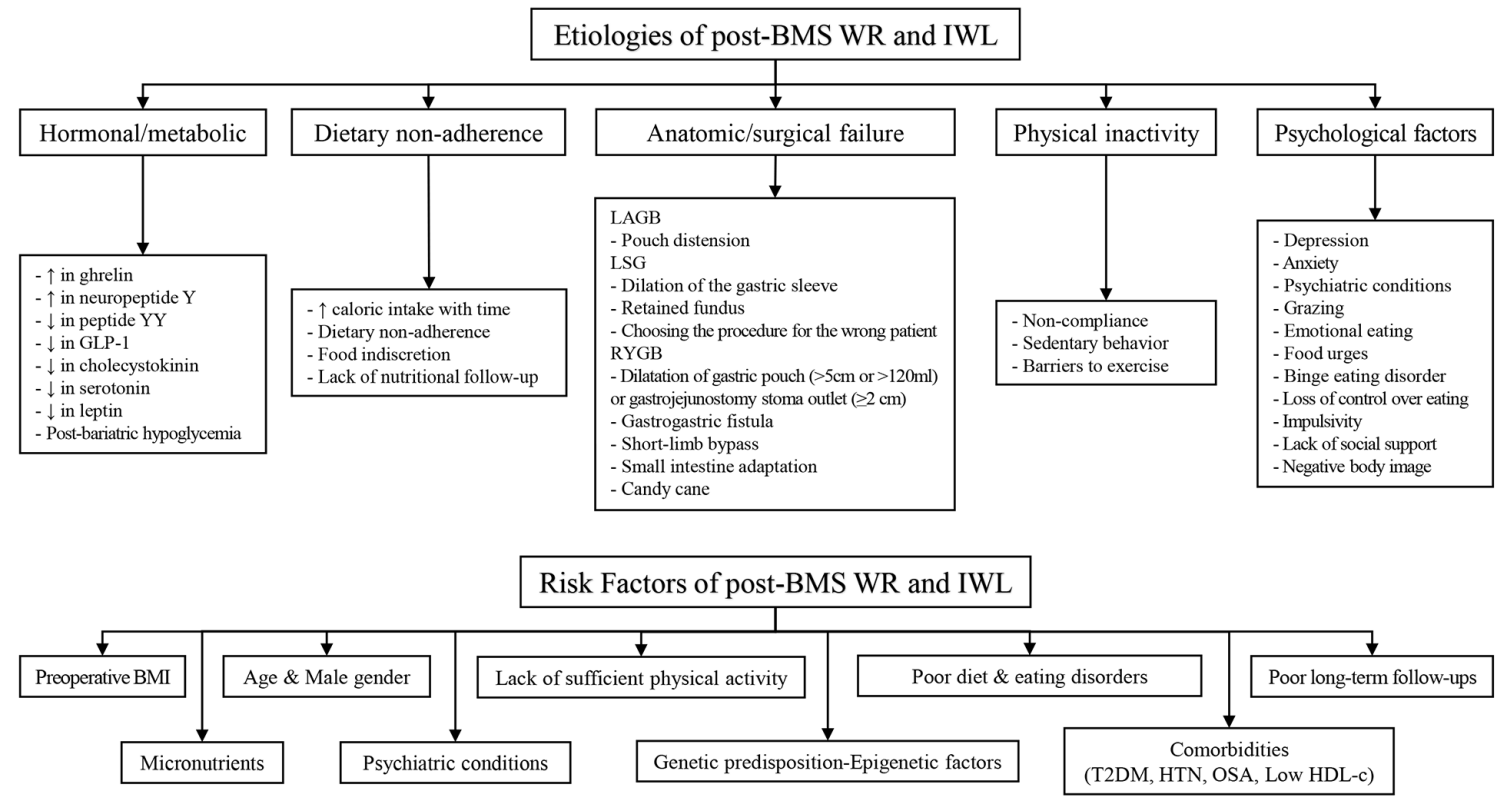
Etiologies and risk factors of insufficient weight loss (IWL) and weight regain (WR) following bariatric metabolic surgery (BMS). IWL, insufficient weight loss; WR, weight regain; BMS, bariatric metabolic surgery; LAGB, laparoscopic adjustable gastric banding; LSG, laparoscopic sleeve gastrectomy; RYGB, roux-en-y gastric bypass; BMI, body mass index; T2DM, type 2 diabetes mellitus; HTN, hypertension; OSA, obstructive sleep apnea; HDL, High-density lipoprotein.

The etiology of post-BMS WR and IWL is multifactorial, and various mechanisms can be attributed to them. These etiologies include five main categories, namely hormonal/metabolic, dietary non-adherence, physical inactivity, psychological factors, and anatomic/surgical failure (Figure 1) (33-46). Anatomical failures have different reasons based on the technique, including pouch distension for the LAGB technique, dilation of the gastric sleeve, retained fundus, and choosing the procedure for the wrong patient for the LSG technique. Particularly in the RYGB technique, the dilatation of the gastric pouch (>5 cm or >120 mL) or gastrojejunostomy stoma outlet (≥2 cm), gastrogastric fistula, short-limb bypass, small intestine adaptation, and candy cane are among the common failure reasons (34, 39, 47). There are many similarities between the etiology and possible RFs, which are discussed below.

### 3.4. Risk Factors

Many RFs have been introduced for suboptimal weight loss and WR, although fewer studies have assessed RFs on weight loss. These RFs include preoperative BMI, male gender, psychiatric conditions, comorbidities, age, and genetic predisposition-epigenetic factors (3, 35). Some modifiable RFs, including unhealthy dietary habits, food intolerance, eating disorders, poor long-term follow-ups, insufficient patient knowledge, and lack of sufficient physical activity, are significantly associated with WR (48) due to their potential effects on loss of appetite control and increased eating frequency (49). Consequently, WR could refer to the metabolic overfeeding process, defined as nutrient excess and positive energy balance, along with diminished energy expenditure and resting metabolic rate.

Reduced metabolic rate results from adaptive thermogenesis following post-bariatric weight loss and alterations in fat mass and lean body mass in the first six months (17, 36). As mentioned earlier, WR might also be a result of changes and imbalances in the gut and adipocyte hormones, which comprise rising ghrelin, neuropeptide Y (NPY), insulin sensitivity, and dropped peptide YY (PYY), cholecystokinin (CCK), glucagon-like peptide 1 (GLP-1), serotonin, and leptin and eventually hypoglycemic, and even insulin resistance (17, 36, 49, 50). Finally, abnormal estrogen levels among women have been implicated in WR (49).

Preoperative BMI: Preoperative BMI is among the most important RFs for post-BMS IWL and WR. In a study by Csendes et al., 85 - 100% of patients after sleeve gastroplasty (SG) with a preoperative BMI ≥40 kg/m^2^ developed WR within 78 - 138 months. However, in the same period, only 3.6 - 38% of patients with preoperative BMI <40 kg/m^2^ regained weight (37). However, in 2012, Livhits et al. conducted a systematic review and discovered that, of the 62 studies examining the association between preoperative BMI and post-surgery weight loss, the majority (37 studies), primarily focusing on EWL after RYGB, discovered a negative association between baseline BMI and weight loss (51). This disparity is most likely caused by earlier research reporting weight loss data as TWL rather than EWL (52).

Psychiatric Conditions: Psychiatric conditions can also play an important role, especially for WR (38). It has been determined that preoperative psychiatric problems are not strongly associated with WR; however, postoperative psychiatric problems are among the strongest RFs and etiologies (38). Eating psychopathology (38), particularly grazing, loss of control over eating, emotional eating, and food urges (40-43), were observed to be substantially related to post-BMS WR. In addition, WR was linked to binge eating in both the short and long term following BMS (53). Impulsivity has been shown to be a key component of disordered eating patterns and obesity, and it can lead to less weight loss results following surgery (44).

Furthermore, a lack of social support and anxiety were linked to less weight loss and higher WR (39). Additionally, the incidence of depressive symptoms was only related to WR in the long term, although the directionality is unknown (53). It has been assumed that negative body image is linked to worse mental health and increased symptoms of depression. Depression symptoms can contribute to problematic eating behaviors, leading to WR (54, 55).

Comorbidities: Comorbidities can also be attributed to IWL and WR, including a significant association between type 2 diabetes mellitus (T2DM) with both IWL and WR (29, 45, 56, 57) and a history of hypertension and obstructive sleep apnea (OSA) with IWL (33, 45).

Gender and age: Male gender is also significantly associated with suboptimal weight loss in studies, even after possible adjustment (29, 46, 58); however, numerous cultural factors might confound this association (9). Age as an RF remains controversial; therefore, some studies have declared old age (age > 60 years) as an RF for IWL and WR post-BMS; nevertheless, some studies have introduced young age as an RF (56, 57). The consistency of this association is weak, and many cultural and other confounding factors might affect these associations (59).

Micronutrients: Micronutrient deficiencies have been recognized as crucial factors affecting the weight management of individuals who have undergone bariatric procedures. These deficiencies can arise due to the altered anatomy of the digestive system, affecting nutrient absorption and impacting various aspects of health, including energy levels, exercise capacity, and overall metabolic function (60). For instance, vitamin B12, vitamin D, and iron deficiency can lead to reduced energy expenditure and compromised fat utilization due to their vital functions in energy metabolism and homeostasis that might contribute to WR. Additionally, inadequate levels of zinc and magnesium could lead to altered appetite signaling, potentially fostering overconsumption and WR; therefore, they can modulate appetite and satiety hormones.

Some micronutrient deficiencies, especially selenium or iodine, might influence thyroid function and overall metabolic activity. A sluggish metabolic rate could facilitate obesity recurrence (61). In addition, micronutrient deficiencies might contribute to the loss of lean muscle mass, which is pivotal for upholding metabolic rate and supporting long-term weight maintenance, resulting in WR. Additionally, it can incite cravings for certain foods, potentially prompting the consumption of calorie-dense, nutrient-poor options and impeding weight loss maintenance endeavors. Diminished lean mass can precipitate a decline in basal metabolic rate, exacerbating the propensity for WR.

### 3.5. Management Strategy

**Figure 2.**
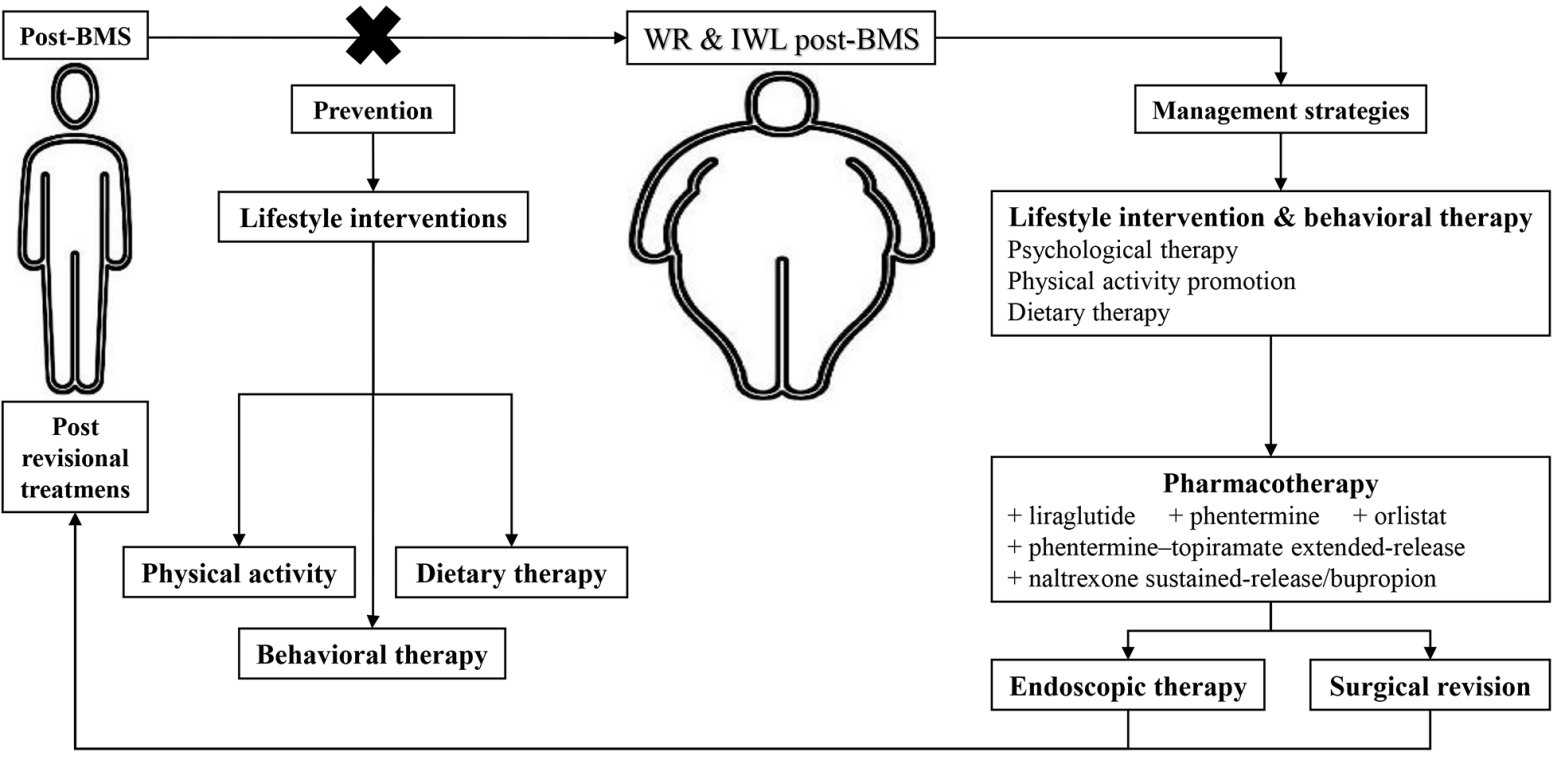
Preventions and management strategies algorithm of post-bariatric metabolic surgery (BMS) insufficient weight loss (IWL) and weight regain (WR). BMS, bariatric metabolic surgery; IWL, insufficient weight loss; WR, weight regain.

Considering the multifactorial etiology and mentioned RFs, treating the IWL and WR following BMS is complex and requires several simultaneous approaches. Firstly, with a comprehensive assessment, all the influential factors in the patient should be examined. Then, a decision should be made based on the patient’s conditions, including dietary patterns, level of physical activity, psychiatric condition, and patient wishes. Post-BMS IWL and WR management strategies include lifestyle intervention, pharmacotherapy, endoscopic therapy, and surgical revision (Figure 2) (3, 6, 17, 62-66).

#### 3.5.1. Lifestyle Interventions

Lifestyle modifications should be fundamental to any management strategy due to their high importance and efficacy (62). The goal is to create a long-term lifestyle based on diet and physical activity (67). As the first principle, a proper, non-judgmental follow-up should be done to avoid losing patients struggling with recovery (62). Lifestyle interventions contain three main areas.

Dietary therapy: Nutrition care is based on face-to-face counseling by a registered dietitian and nutrition prescription, which forms a principal part of structured dietary intervention for weight control (3). In this regard, it has been proposed that dietary intake of specific food items and groups can make the normal balance among gastrointestinal (GI) hormones as described above. For example, ingestion of anti-inflammatory foods, which include berries, fatty fish, broccoli, avocados, green tea, mushrooms, grapes, turmeric, extra virgin olive oil, dark chocolate, cocoa, tomatoes, cherries, and peppers due to their high phytochemicals and antioxidants containing, can be effectively helpful to improve leptin resistance and enhance insulin sensitivity and rising CCK. Additionally, it is also recommended to individuals with WR that the consumption of inflammatory food compounds containing unsaturated fats, junk foods, refined carbohydrates, fried foods, sugar-sweetened drinks, processed meats, soft beverages, and even healthy fats be restricted in order to regulate appetite via leptin and CCK. Researchers noted that dietary fiber intake, high protein diets derived from animal or plant sources, probiotics, and low carbohydrate diets could increment PYY levels and drop NPY in this group. To elevate GLP-1 levels, it has been offered to consume excellent protein sources, including fish, whey protein, and yogurt, an anti-inflammation diet, leafy green vegetables (spinach and kale), and probiotics.

In addition, limitations intake in terms of sweetened drinks, including fruit juices, sports (energic) drinks, sweetened waters, regular soda (not sugar-free ones), coffee and tea beverages, along with added sugars and high-fructose corn syrup, because following such foods-eating impaired proper ghrelin response, have been advised to reduce ghrelin, which can act as the hunger hormone (49). It has been well recognized in the literature that the balance of estrogen in women can be made by having fiber-rich sources, flax seeds, and cruciferous vegetables, such as arugula, bok choy, broccoli, brussels sprouts, and cabbage (49). As a result, a registered dietitian should recommend and plan a high-protein, low-glycemic index, lower caloric-density foods (raw fruits and vegetables), and low-fat content diet for patients who experience WR or avoid weight gain (17).

Another good point to mention is that nutritionists should pay much attention to nutritional problems in the nutritional screening during follow-up clinic visits because it is well-recognized that several nutritional deficiencies, including increased parathyroid hormone, decreased pre-albumin, and deficiencies of vitamin B12, vitamin A, zinc, iron, vitamin D, and most importantly protein, might be present in patients with IWL and WR (68). As a result, right after the liquid feeding step after BMS, a protein supplement with a dosage of 1 - 1.5 g/kg of ideal weight or 60-120 g/day is necessary to maintain fat-free mass or slow lean muscle loss (17, 69). The bariatric plate model and bariatric traffic light system might be used to guide patients about their diet (70).

Physical activity promotion: Due to the lack of guidelines on the amount of physical activity after BMS, it is generally recommended to exercise moderately to vigorously for 150 - 250 minutes/week with the help of a physical trainer as an essential component of weight management regimens to prevent WR and > 300 minutes/week to maintain weight loss (71). Thirty days after BMS, regular exercise, including weight training and aerobic activity, is advised (63).

Psychological and behavioral therapy: Psychotherapeutic interventions and support groups significantly affect post-BMS weight loss (64). Effective, significantly associated psychological and behavioral treatments include cognitive behavioral therapy, remote acceptance-based behavioral intervention, and lifestyle counseling (3, 64). Furthermore, mindfulness-based approaches, dialectical behavioral therapy, and various therapeutic strategies can be employed. According to the data, post-operative behavioral therapies are currently the most promising for enhancing short-term weight reduction outcomes in bariatric surgery patients (72).

#### 3.5.2. Pharmacotherapy

Several antiobesity medications have decreased hunger, promoted satiety, and stopped post-BMS WR (6). Among the available drugs, liraglutide, phentermine, phentermine-topiramate extended-release, orlistat, and naltrexone sustained-release/bupropion are the United States Food and Drug Administration (FDA)-approved, and metformin, topiramate, zonisamide, and bupropion are used off label (6, 73). Nevertheless, pharmacotherapy can help reduce weight more effectively by adding to lifestyle interventions and behavioral therapies. However, these antiobesity medications should be prescribed at the weight plateau and not after the WR (73). Few retrospective studies have been conducted on antiobesity medications in the revisional treatment of post-BMS IWL and WR, raising a great need for clinical trials.

It is suggested to use guidelines for treatment and dosage for obese patients without surgery. Starting with the lowest dose possible and adjusting as necessary is the best practice for treatment. When individual active substances are ineffectual, combination treatments might be used; nevertheless, only seasoned practitioners should consider this alternative (62).

#### 3.5.3. Endoscopic Therapy

Esophagogastroduodenoscopy is an essential evaluation of post-BMS IWL and WR. This diagnostic procedure assesses altered anatomy and surgical complications (65). The recommended endoscopic revision therapies vary depending on the type of surgery performed. Suggesting a superior technique over the others is still being determined. Further studies are needed to determine the optimal technique with the highest safety and efficacy of endoscopic revisional treatments.

Post-RYGB WR: Larger gastro-jejunal (GJ) stoma size over time is a common anatomical change after RYGB significantly associated with WR (74). Due to the difficulty of the dissection and the high risk of complications, endoscopic revisions are recommended, especially in patients with GJ anastomosis dilatation >15 mm or gastric pouch dilatation >5 cm in length (75). Several techniques have been recommended, including sclerotherapy, argon plasma coagulation (APC), endoluminal reduction of the GJ anastomosis and/or pouch with plication or suturing with/without APC, and endoscopic submucosal dissection with APC and suturing (75).

Post-gastric sleeve WR: Endoscopic approaches are mainly performed to treat WR in post-RYGB patients. However, due to the recent rise of SG, endoscopic suturing and plication are becoming popular in post-SG patients with WR (76). A retrospective, multicenter study that included patients who underwent revisional endoscopic SG following post-BMS WR reported that all included patients achieved ≥25% EWL with no serious adverse events (75).

### 3.5.4 Surgical Revision

The last line for the treatment of IWL and WR post-BMS is revisional surgeries. There are few trial studies on this subject; however, in a recent systematic review study, six techniques of surgeries, including endoscopic gastroplasty (ESG), re-sleeve gastrectomy, RYGB, OAGB, single-anastomosis duodeno-ileal bypass (SADI), and duodenal switch (DS), were investigated in post-SG patients. This study reported that all BMS techniques were successful. One-anastomosis gastric bypass had an excellent balance between weight loss and complications; the least efficient BMS was ESG, and SADI and DS significantly increased complications’ incidence (66, 77, 78).

After primary BMS, patients with IWL or WR have modest weight loss with adjunctive pharmacotherapy. Conversely, revisional BMS is an effective treatment for IWL and WR (79). This study also reports that TWL is significantly higher in the IWL group than in the WR group in revisional BMS, demonstrating that surgical treatment in IWL patients is more effective than WR (79). Finally, the choice of procedure depends on the patient’s characteristics and the surgeon’s expertise. Further research from prospective randomized controlled trials (RCTs) is needed to substantiate these findings.

### 3.6. Prevention

There are few studies regarding prevention, mainly concentrating on enhancing weight reduction results and associated lifestyle-related behaviors. These studies either failed to find any effects or found relatively minor ones (80). In any case, behavioral and psychotherapeutic interventions, dietary therapy, and physical activity therapy are the essential components of prevention, just as they are in treatment (Figure 2) (64, 80, 81). Naturally, there are controversial reports in this regard (80). According to a systematic review by Rudolph and Hilbert patients had higher WL throughout cognitive behavioral therapy and group support following RYGB (64). Additionally, consulting a nutritionist for 15 minutes every week for the first four postoperative months resulted in significantly more loss of TWL (81). Further clinical studies, particularly RCTs, are required to pinpoint efficient preventative tactics.

## 4. Conclusions

Bariatric metabolic surgery has been identified as the most effective and long-lasting method for reducing body weight. Insufficient weight loss and weight regain following BMS, which are the usual causes to qualify for revisional BMS, might happen in a variable percentage of patients. Defining a precise definition for IWL/WR can help determine the prevalence, RFs, and etiology. In the same direction, the prevalence of IWL and WR is still being determined due to low follow-up rates, the type of BMS performed, and various definitions.

Although the exact etiology of post-BMS IWL and WR is still unknown, several mechanisms, including hormonal/metabolic, dietary non-adherence, physical inactivity, mental health, and anatomic surgical failure, are thought to be potential etiologies. The most important RFs have been preoperative BMI, male gender, psychiatric conditions, comorbidities, age, sticking to a poor diet, eating disorders, poor long-term follow-ups, lack of sufficient physical activity, micronutrients, and genetic predisposition-epigenetic factors.

The management strategy for post-BMS IWL and WR is multidisciplinary. The principle and basis of treatment are lifestyle interventions. Pharmacotherapy can be added to these treatments if needed. In the last treatment line, different techniques of endoscopic surgery and revisional surgery can be used based on the type of anatomy, the patient’s characteristics, and the surgeon’s expertise. Finally, behavioral and psychotherapeutic interventions, dietary therapy, and physical activity therapy are the essential components of prevention, just as they are in treatment. However, an improved understanding of the hormonal, psychological, behavioral, and surgical factors causing WR/IWL might offer a more reliable body of evidence on which suitable preventative and management measures could be based. Additionally, the management of WR/IWL urgently requires proof of efficacy in RCTs for cost-effective medication in conjunction with lifestyle modification.

## References

[1] Courcoulas AP, Yanovski SZ, Bonds D, Eggerman TL, Horlick M, Staten MA (2014). Long-term outcomes of bariatric surgery: a National Institutes of Health symposium.. JAMA Surg..

[2] Andalib A, Alamri H, Almuhanna Y, Bouchard P, Demyttenaere S, Court O (2021). Short-term outcomes of revisional surgery after sleeve gastrectomy: a comparative analysis of re-sleeve, Roux en-Y gastric bypass, duodenal switch (Roux en-Y and single-anastomosis).. Surg Endosc..

[3] El Ansari W, Elhag W (2021). Weight Regain and Insufficient Weight Loss After Bariatric Surgery: Definitions, Prevalence, Mechanisms, Predictors, Prevention and Management Strategies, and Knowledge Gaps-a Scoping Review.. Obes Surg..

[4] Corcelles R, Boules M, Froylich D, Hag A, Daigle CR, Aminian A (2016). Total Weight Loss as the Outcome Measure of Choice After Roux-en-Y Gastric Bypass.. Obes Surg..

[5] Rogula TG (2015). Weight regain after bariatric surgery—how should it be defined?. Population..

[6] Stanford FC, Alfaris N, Gomez G, Ricks ET, Shukla AP, Corey KE (2017). The utility of weight loss medications after bariatric surgery for weight regain or inadequate weight loss: A multi-center study.. Surg Obes Relat Dis..

[7] Uittenbogaart M, de Witte E, Romeijn MM, Luijten A, van Dielen FMH, Leclercq WKG (2020). Primary and Secondary Nonresponse Following Bariatric Surgery: a Survey Study in Current Bariatric Practice in the Netherlands and Belgium.. Obes Surg..

[8] Grover BT, Morell MC, Kothari SN, Borgert AJ, Kallies KJ, Baker MT (2019). Defining Weight Loss After Bariatric Surgery: a Call for Standardization.. Obes Surg..

[9] Bastos EC, Barbosa EM, Soriano GM, dos Santos EA, Vasconcelos SM (2013). Determinants of weight regain after bariatric surgery.. Arq Bras Cir Dig..

[10] Lauti M, Lemanu D, Zeng ISL, Su'a B, Hill AG, MacCormick AD (2017). Definition determines weight regain outcomes after sleeve gastrectomy.. Surg Obes Relat Dis..

[11] Liu SY, Wong SK, Lam CC, Yung MY, Kong AP, Ng EK (2015). Long-term Results on Weight Loss and Diabetes Remission after Laparoscopic Sleeve Gastrectomy for A Morbidly Obese Chinese Population.. Obes Surg..

[12] Brethauer SA, Kim J, El Chaar M, Papasavas P, Eisenberg D, Rogers A (2015). Standardized outcomes reporting in metabolic and bariatric surgery.. Obes Surg..

[13] Kermansaravi M, Davarpanah Jazi AH, Shahabi Shahmiri S, Eghbali F, Valizadeh R, Rezvani M (2021). Revision procedures after initial Roux-en-Y gastric bypass, treatment of weight regain: a systematic review and meta-analysis.. Updates Surg..

[14] Himpens J, Coromina L, Verbrugghe A, Cadiere GB (2012). Outcomes of revisional procedures for insufficient weight loss or weight regain after Roux-en-Y gastric bypass.. Obes Surg..

[15] Lauti M, Kularatna M, Hill AG, MacCormick AD (2016). Weight Regain Following Sleeve Gastrectomy-a Systematic Review.. Obes Surg..

[16] Voorwinde V, Steenhuis IHM, Janssen IMC, Monpellier VM, van Stralen MM (2020). Definitions of Long-Term Weight Regain and Their Associations with Clinical Outcomes.. Obes Surg..

[17] Istfan NW, Lipartia M, Anderson WA, Hess DT, Apovian CM (2021). Approach to the Patient: Management of the Post-Bariatric Surgery Patient With Weight Regain.. J Clin Endocrinol Metab..

[18] King WC, Hinerman AS, Belle SH, Wahed AS, Courcoulas AP (2018). Comparison of the Performance of Common Measures of Weight Regain After Bariatric Surgery for Association With Clinical Outcomes.. JAMA..

[19] Roth AE, Thornley CJ, Blackstone RP (2020). Outcomes in Bariatric and Metabolic Surgery: an Updated 5-Year Review.. Curr Obes Rep..

[20] Thomas DD, Anderson WA, Apovian CM, Hess DT, Yu L, Velazquez A (2019). Weight Recidivism After Roux-en-Y Gastric Bypass Surgery: An 11-Year Experience in a Multiethnic Medical Center.. Obesity (Silver Spring)..

[21] da Silva FB, Gomes DL, de Carvalho KM (2016). Poor diet quality and postoperative time are independent risk factors for weight regain after Roux-en-Y gastric bypass.. Nutrition..

[22] Dos Rodrigues LS, de Vasconcelos PHC, Gomes DL (2021). Weight regain and eating behavior in physically active and inactive women after 24 months of bariatric surgery.. Eat Weight Disord..

[23] Nedelcu M, Khwaja HA, Rogula TG (2016). Weight regain after bariatric surgery-how should it be defined?. Surg Obes Relat Dis..

[24] Nicolau J, Simo R, Sanchis P, Ayala L, Fortuny R, Rivera R (2017). Effects of depressive symptoms on clinical outcomes, inflammatory markers and quality of life after a significant weight loss in a bariatric surgery sample.. Nutr Hosp..

[25] Conceicao EM, Mitchell JE, Pinto-Bastos A, Arrojado F, Brandao I, Machado PPP (2017). Stability of problematic eating behaviors and weight loss trajectories after bariatric surgery: a longitudinal observational study.. Surg Obes Relat Dis..

[26] Sousa P, Bastos AP, Venancio C, Vaz AR, Brandao I, Costa JM (2014). [Understanding depressive symptoms after bariatric surgery: the role of weight, eating and body image].. Acta Med Port..

[27] Sjostrom L, Lindroos AK, Peltonen M, Torgerson J, Bouchard C, Carlsson B (2004). Lifestyle, diabetes, and cardiovascular risk factors 10 years after bariatric surgery.. N Engl J Med..

[28] Clapp B, Wynn M, Martyn C, Foster C, O'Dell M, Tyroch A (2018). Long term (7 or more years) outcomes of the sleeve gastrectomy: a meta-analysis.. Surg Obes Relat Dis..

[29] Courcoulas AP, King WC, Belle SH, Berk P, Flum DR, Garcia L (2018). Seven-Year Weight Trajectories and Health Outcomes in the Longitudinal Assessment of Bariatric Surgery (LABS) Study.. JAMA Surg..

[30] Conceicao E, Mitchell JE, Vaz AR, Bastos AP, Ramalho S, Silva C (2014). The presence of maladaptive eating behaviors after bariatric surgery in a cross sectional study: importance of picking or nibbling on weight regain.. Eat Behav..

[31] Homan J, Betzel B, Aarts EO, van Laarhoven KJ, Janssen IM, Berends FJ (2015). Secondary surgery after sleeve gastrectomy: Roux-en-Y gastric bypass or biliopancreatic diversion with duodenal switch.. Surg Obes Relat Dis..

[32] Abdulrazzaq S, Elhag W, El Ansari W, Mohammad AS, Sargsyan D, Bashah M (2020). Is Revisional Gastric Bypass as Effective as Primary Gastric Bypass for Weight Loss and Improvement of Comorbidities?. Obes Surg..

[33] Cadena-Obando D, Ramirez-Renteria C, Ferreira-Hermosillo A, Albarran-Sanchez A, Sosa-Eroza E, Molina-Ayala M (2020). Are there really any predictive factors for a successful weight loss after bariatric surgery?. BMC Endocr Disord..

[34] Yarigholi F, Bahardoust M, Mosavari H, Tehrani FM, Gholizadeh H, Shahmiri SS (2022). Predictors of Weight Regain and Insufficient Weight Loss According to Different Definitions After Sleeve Gastrectomy: a Retrospective Analytical Study.. Obes Surg..

[35] Gutt S, Schraier S, Gonzalez Bagnes MF, Yu M, Gonzalez CD, Di Girolamo G (2019). Long-term pharmacotherapy of obesity in patients that have undergone bariatric surgery: pharmacological prevention and management of body weight regain.. Expert Opin Pharmacother..

[36] Johnson Stoklossa C, Atwal S (2013). Nutrition care for patients with weight regain after bariatric surgery.. Gastroenterol Res Pract..

[37] Csendes A, Burgos AM, Martinez G, Figueroa M, Castillo J, Diaz JC (2018). Loss and Regain of Weight After Laparoscopic Sleeve Gastrectomy According to Preoperative BMI : Late Results of a Prospective Study (78-138 months) with 93% of Follow-Up.. Obes Surg..

[38] Mauro M, Papelbaum M, Brasil MAA, Carneiro JRI, Coutinho ESF, Coutinho W (2019). Is weight regain after bariatric surgery associated with psychiatric comorbidity? A systematic review and meta-analysis.. Obes Rev..

[39] Athanasiadis DI, Martin A, Kapsampelis P, Monfared S, Stefanidis D (2021). Factors associated with weight regain post-bariatric surgery: a systematic review.. Surg Endosc..

[40] Pizato N, Botelho PB, Goncalves VSS, Dutra ES, de Carvalho KMB (2017). Effect of Grazing Behavior on Weight Regain Post-Bariatric Surgery: A Systematic Review.. Nutrients..

[41] Conceicao E, Bastos AP, Brandao I, Vaz AR, Ramalho S, Arrojado F (2014). Loss of control eating and weight outcomes after bariatric surgery: a study with a Portuguese sample.. Eat Weight Disord..

[42] Bakr AA, Fahmy MH, Elward AS, Balamoun HA, Ibrahim MY, Eldahdoh RM (2019). Analysis of Medium-Term Weight Regain 5 Years After Laparoscopic Sleeve Gastrectomy.. Obes Surg..

[43] Odom J, Zalesin KC, Washington TL, Miller WW, Hakmeh B, Zaremba DL (2010). Behavioral predictors of weight regain after bariatric surgery.. Obes Surg..

[44] Sarwer DB, Allison KC, Wadden TA, Ashare R, Spitzer JC, McCuen-Wurst C (2019). Psychopathology, disordered eating, and impulsivity as predictors of outcomes of bariatric surgery.. Surg Obes Relat Dis..

[45] de Raaff CA, Coblijn UK, de Vries N, Heymans MW, van den Berg BT, van Tets WF (2016). Predictive Factors for Insufficient Weight Loss After Bariatric Surgery: Does Obstructive Sleep Apnea Influence Weight Loss?. Obes Surg..

[46] Melton GB, Steele KE, Schweitzer MA, Lidor AO, Magnuson TH (2008). Suboptimal weight loss after gastric bypass surgery: correlation of demographics, comorbidities, and insurance status with outcomes.. J Gastrointest Surg..

[47] Mahmoudieh M, Keleidari B, Salimi M, Sayadi M, Shahabi S, Sheikhbahaei E (2019). The two different biliopancreatic limb lengths for roux-en-Y gastric bypass.. Obesity Medicine..

[48] Kaouk L, Hsu AT, Tanuseputro P, Jessri M (2019). Modifiable factors associated with weight regain after bariatric surgery: a scoping review.. F1000Res..

[49] Kamali Ardekani M, Lacy VA, Eshghjoo S, Anbara T (2022). Possible Weight Regain Managements after Bariatric Surgery.. Obesity and metabolism..

[50] Keleidari B, Mohammadi Mofrad R, Shahabi Shahmiri S, Sanei MH, Kolahdouzan M, Sheikhbahaei E (2018). The Impacts of Gastroileostomy Rat Model on Glucagon-like Peptide-1: a Promising Model to Control Type 2 Diabetes Mellitus.. Obes Surg..

[51] Livhits M, Mercado C, Yermilov I, Parikh JA, Dutson E, Mehran A (2012). Preoperative predictors of weight loss following bariatric surgery: systematic review.. Obes Surg..

[52] Shukla AP, He D, Saunders KH, Andrew C, Aronne LJ (2018). Current concepts in management of weight regain following bariatric surgery.. Expert Rev Endocrinol Metab..

[53] Freire CC, Zanella MT, Segal A, Arasaki CH, Matos MIR, Carneiro G (2021). Associations between binge eating, depressive symptoms and anxiety and weight regain after Roux-en-Y gastric bypass surgery.. Eat Weight Disord..

[54] Ortega J, Fernandez-Canet R, Alvarez-Valdeita S, Cassinello N, Baguena-Puigcerver MJ (2012). Predictors of psychological symptoms in morbidly obese patients after gastric bypass surgery.. Surg Obes Relat Dis..

[55] Geerts MM, van den Berg EM, van Riel L, Peen J, Goudriaan AE, Dekker JJM (2021). Behavioral and psychological factors associated with suboptimal weight loss in post-bariatric surgery patients.. Eat Weight Disord..

[56] Paul L, van der Heiden C, Hoek HW (2017). Cognitive behavioral therapy and predictors of weight loss in bariatric surgery patients.. Curr Opin Psychiatry..

[57] Al-Khyatt W, Ryall R, Leeder P, Ahmed J, Awad S (2017). Predictors of Inadequate Weight Loss After Laparoscopic Gastric Bypass for Morbid Obesity.. Obes Surg..

[58] Pellitero S, Perez-Romero N, Martinez E, Granada ML, Moreno P, Balibrea JM (2015). Baseline circulating ghrelin does not predict weight regain neither maintenance of weight loss after gastric bypass at long term.. Am J Surg..

[59] Monaco-Ferreira DV, Leandro-Merhi VA (2017). Weight Regain 10 Years After Roux-en-Y Gastric Bypass.. Obes Surg..

[60] Allied Health Sciences Section Ad Hoc Nutrition C, Aills L, Blankenship J, Buffington C, Furtado M, Parrott J (2008). ASMBS Allied Health Nutritional Guidelines for the Surgical Weight Loss Patient.. Surg Obes Relat Dis..

[61] Parrott J, Frank L, Rabena R, Craggs-Dino L, Isom KA, Greiman L (2017). American Society for Metabolic and Bariatric Surgery Integrated Health Nutritional Guidelines for the Surgical Weight Loss Patient 2016 Update: Micronutrients.. Surg Obes Relat Dis..

[62] Velapati SR, Shah M, Kuchkuntla AR, Abu-Dayyeh B, Grothe K, Hurt RT (2018). Weight Regain After Bariatric Surgery: Prevalence, Etiology, and Treatment.. Curr Nutr Rep..

[63] Sjostrom L (2013). Review of the key results from the Swedish Obese Subjects (SOS) trial - a prospective controlled intervention study of bariatric surgery.. J Intern Med..

[64] Rudolph A, Hilbert A (2013). Post-operative behavioural management in bariatric surgery: a systematic review and meta-analysis of randomized controlled trials.. Obes Rev..

[65] de Moura DTH, da Ponte-Neto AM, Hathorn KE, do Monte Junior ES, Baptista A, Ribeiro IB (2020). Novel Endoscopic Management of a Chronic Gastro-Gastric Fistula Using a Cardiac Septal Defect Occluder.. Obes Surg..

[66] Franken RJ, Sluiter NR, Franken J, de Vries R, Souverein D, Gerdes VEA (2022). Treatment Options for Weight Regain or Insufficient Weight Loss After Sleeve Gastrectomy: a Systematic Review and Meta-analysis.. Obes Surg..

[67] Wadden TA, Butryn ML, Hong PS, Tsai AG (2014). Behavioral treatment of obesity in patients encountered in primary care settings: a systematic review.. JAMA..

[68] Bradley LE, Forman EM, Kerrigan SG, Goldstein SP, Butryn ML, Thomas JG (2017). Project HELP: a Remotely Delivered Behavioral Intervention for Weight Regain after Bariatric Surgery.. Obes Surg..

[69] Lupoli R, Lembo E, Saldalamacchia G, Avola CK, Angrisani L, Capaldo B (2017). Bariatric surgery and long-term nutritional issues.. World J Diabetes..

[70] Cambi MPC, Baretta GAP (2018). Bariatric Diet Guide: Plate Model Template for Bariatric Surgery Patients.. Arq Bras Cir Dig..

[71] Wefers JF, Woodlief TL, Carnero EA, Helbling NL, Anthony SJ, Dubis GS (2017). Relationship among physical activity, sedentary behaviors, and cardiometabolic risk factors during gastric bypass surgery-induced weight loss.. Surg Obes Relat Dis..

[72] David LA, Sijercic I, Cassin SE (2020). Preoperative and post-operative psychosocial interventions for bariatric surgery patients: A systematic review.. Obes Rev..

[73] Nor Hanipah Z, Nasr EC, Bucak E, Schauer PR, Aminian A, Brethauer SA (2018). Efficacy of adjuvant weight loss medication after bariatric surgery.. Surg Obes Relat Dis..

[74] Abu Dayyeh BK, Lautz DB, Thompson CC (2011). Gastrojejunal stoma diameter predicts weight regain after Roux-en-Y gastric bypass.. Clin Gastroenterol Hepatol..

[75] Cambi MPC, Baretta GAP, Magro DO, Boguszewski CL, Ribeiro IB, Jirapinyo P (2021). Multidisciplinary Approach for Weight Regain-how to Manage this Challenging Condition: an Expert Review.. Obes Surg..

[76] de Miranda Neto AA, de Moura DTH, Ribeiro IB, Khan A, Singh S, da Ponte Neto AM (2020). Efficacy and Safety of Endoscopic Sleeve Gastroplasty at Mid Term in the Management of Overweight and Obese Patients: a Systematic Review and Meta-Analysis.. Obes Surg..

[77] Keleidari B, Mahmoudieh M, Shahabi S, Sheikhbahaei E, Rezaei M, Sayadi M (2020). Reversing One-Anastomosis Gastric Bypass Surgery due to Severe and Refractory Hypoalbuminemia.. World J Surg..

[78] Mahmoudieh M, Keleidari B, Hadipour P, Sheikhbahaei E, Chang AR, Ramtin S (2021). Comparative Effectiveness of Roux-en-Y Gastric Bypass vs. One Anastomosis Gastric Bypass on Kidney Function.. Obes Surg..

[79] Dharmaratnam VM, Lim E, Eng A, Chan WH, Tan HC, Ho E (2022). Revisional Surgery or Pharmacotherapy for Insufficient Weight Loss and Weight Regain After Primary Bariatric Procedure: a Descriptive Study.. Obes Surg..

[80] Kushner RF, Sorensen KW (2015). Prevention of Weight Regain Following Bariatric Surgery.. Curr Obes Rep..

[81] Sarwer DB, Moore RH, Spitzer JC, Wadden TA, Raper SE, Williams NN (2012). A pilot study investigating the efficacy of postoperative dietary counseling to improve outcomes after bariatric surgery.. Surg Obes Relat Dis..

